# Laser writing of plasmonic catalytic microchannels on UiO-66 layer

**DOI:** 10.1039/d5nr05068e

**Published:** 2026-02-23

**Authors:** Alina Gorbunova, Swagato Sarkar, Dmitry Kogolev, Wanderson Ferraz do Valle, Markus Ostermann, Thomas Schachinger, Hradil Klaudia, Alexey Ivanov, Markus Valtiner, Pavel S. Postnikov, Olga Guselnikova

**Affiliations:** a Research School of Chemistry & Applied Biomedical Technology, Tomsk Polytechnic University Tomsk Russia postnikov@tpu.ru; b Institute of Applied Physics, Technical University of Vienna Vienna Austria olga.guselnikova@tuwien.ac.at; c Leibniz-Institut für Polymerforschung Dresden e.V. Hohe Str. 6 01069 Dresden Germany; d Institute of Radiopharmaceutical Cancer Research, Helmholtz-Zentrum Dresden-Rossendorf (HZDR), Bautzner Landstraße 400 01328 Dresden Germany; e University Service Centre for Transmission Electron Microscopy, TU Wien Vienna Austria; f X-Ray Center, TU Wien Vienna Austria; g N.N. Vorozhtsov Institute of Organic Chemistry Pr. Ak. Lavrentieva 9 Novosibirsk 630090 Russia

## Abstract

Plasmon-assisted catalysis offers an attractive route for solar-to-chemical energy conversion, yet its practical integration into scalable flow systems remains limited by complex fabrication steps and poor nanoparticle stability. Here, we report a one-step laser-assisted method for simultaneously forming gold nanoparticles (Au NPs) and patterning microchannels on metal-organic framework (MOF)-functionalized polyethylene terephthalate (PET) substrates derived from waste plastic. Using a 405 nm laser, the *in situ* reduction of HAuCl_4_ within a UiO-66 matrix deposited on PET enables homogeneous Au NPs formation (≈5.4 nm) and precise microchannel definition without the need for additional reagents or post-processing. The resulting PET@Au composites exhibit strong plasmon resonance at 550 nm and catalytic activity for the degradation of methylene blue under visible light, confirming their functional performance. This approach combines plasmonic functionality, low-cost materials, and sustainable processing into a scalable platform for the development of integrated microfluidic and photocatalytic devices.

## Abstract 2

Plasmonic microchannels are patterned by laser-induced *in situ* Au NPs reduction on MOF-functionalized PET for reducing fabrication steps and costs towards plasmon-active microfluidic chips design.

## Graphical abstract

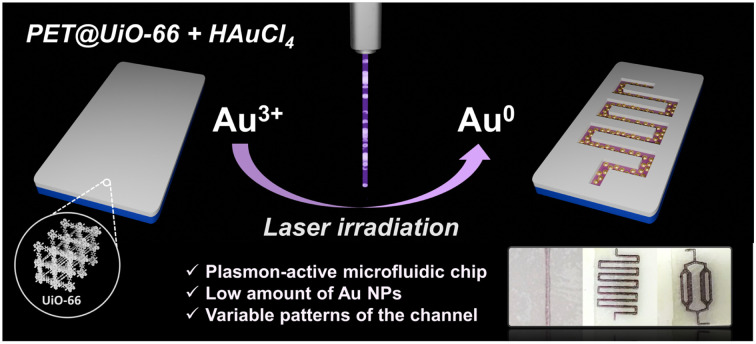

## Introduction

Plasmon-assisted solar-to-chemical energy conversion has emerged as a promising alternative to replace existing non-renewable energy sources.^1^ Plasmon-active nanoparticles (PNPs) based on the noble metals (Au, Ag) excite collective oscillation of the electrons under light irradiation, which can be used for catalytic applications.^2^ Recent studies have further demonstrated that localized surface plasmon resonances can facilitate efficient charge transfer and generate electromagnetic hot-spots, thereby driving enhanced photocatalytic activity in hybrid plasmonic architectures.^3–5^ However, translating the fundamental principles of plasmonic catalysis into scalable industrial processes remains a significant challenge. One viable pathway is the transition from conventional batch or flask-based systems to continuous-flow microfluidic devices.^6,7^ They provide enhanced light–matter interaction, efficient mass and heat transfer, and improved reaction control^8,9^ due to a high surface-to-reactant ratio and reduced consumption of expensive noble metal NPs. However, a major obstacle for catalytic applications is the incorporation of PNPs into flow systems. When PNPs are circulated in microfluidic devices as colloidal suspensions, they tend to agglomerate and adhere to the channel walls (PDMS/glass), which diminishes catalytic reproducibility and long-term stability. The uncontrolled aggregation leads to local electromagnetic field hot-spot loss, whereas excessive adhesion to PDMS or glass surfaces reduces the active particle concentration and changes residence times.^6^

To overcome these limitations, PNPs can be integrated into microfluidic devices, either within channels or on solid substrates, thereby enhancing stability and reusability.^7^ Incorporation into channels could be achieved using template-assisted self-assembly of preformed NPs^10,11^ or *in situ* chemical reduction of metal precursor^12–15^ methods. Nevertheless, these methods often exhibit poor adhesion, non-uniform deposition, and leaching of PNPs during operation. For example, Pellejero *et al.* reported that Au@POM/TiO_2_ coatings transferred during PDMS casting suffered from partial detachment,^10^ while Castedo *et al.* observed cracking and peeling of Au/TiO_2_ layers under prolonged flow.^14^*In situ* reduction within PDMS channels led to uncontrolled aggregation and rough coatings,^12^ and direct substrate growth required APTES adhesion layers to prevent delamination.^13^ Lithographic deposition on glass or F-doped SnO_2_ (FTO) substrates ensures PNPs adhesion,^16^ still, it involves multiple fabrication steps (spin-coating, sputtering, annealing, and laser treatment), thereby increasing the cost and complexity. On the contrary, laser-induced deposition enables the direct reduction of metal precursors under localized irradiation, providing precise control over the size, shape, and spatial distribution of PNPs (Au, Ag, Pd).^17,18^ A notable example is the light-induced protrusion of Au nanowires from Au-ion doped TiO_2_ film reported by Ding T. *et al*.^19^ Irradiation with a continuous-wave laser triggers the reduction and nucleation of Au(0), leading to the vertical growth of nanowires directly from a solid-state substrate. Unlike multi-step lithographic or chemical reduction methods, such a laser-assisted process offers direct patterning and clean fabrication without additional reagents or post-processing.^20^

In this work, we employ a single-step laser treatment that simultaneously enables the formation of Au NPs and microchannel patterning, providing a fast and scalable route to integrated plasmonic devices. As a low-cost alternative to conventional solid substrates, we use waste PET films coated with an MOF layer. Building on our previous findings,^21^ PET can be functionalized with UiO-66, in which terephthalic acid (a PET-derived monomer) forms a porous coating that can incorporate Au precursors. Upon 405 nm laser irradiation, the localized laser-induced reduction drives the *in situ* Au NPs formation within the transformed MOF matrix while defining the microchannel geometry. Moreover, this strategy reduces the amount of Au precursor and uses waste polymer as an initial substrate, offering a perspective for the development of plasmon-active materials in the microfluidics field.

## Results and discussion

### Laser-induced *in situ* formation of Au NPs in MOF-functionalized PET

**Fig. 1:**
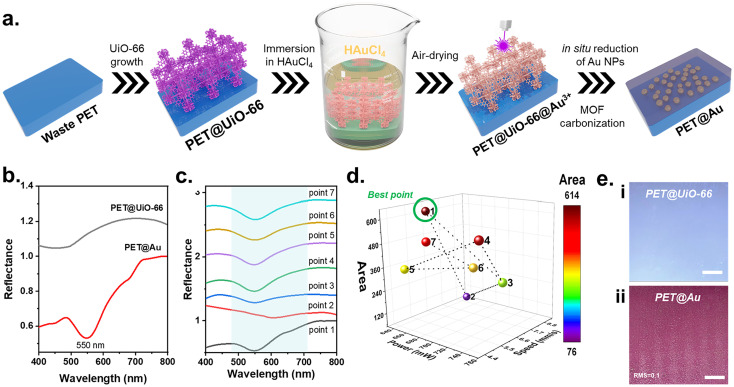
(a) Strategy of the laser-induced *in situ* Au NPs reduction; (b) UV-Vis reflectance spectra of PET@UiO-66 and PET@Au, confirming plasmon resonance at 550 nm; (c) series of UV-Vis spectra recorded under different laser powers and scanning speeds during Nelder–Mead optimization; (d) three-dimensional response surface of the Nelder-Mead optimization showing the dependence of plasmon peak integrated area on laser parameters; (d) optical images of material: (i) PET@UiO-66 and (ii) laser reduced Au NPs on PET@UiO-66, scale bar is 2.5 mm.

Inspired by the laser-induced Au NPs reduction methods,^17^ we began our study by exploring the possibility of using low-cost PET as a substrate for *in situ* NPs formation and control patterning. Here, we used previously reported PET@MOF^21^ to fabricate plasmonic Au NPs within a low-cost PET substrate. While previous studies^22^ have extensively explored MOF-mediated synthesis of metal NPs *via* thermal or chemical reduction routes, our work introduces a previously unreported approach that integrates laser-assisted *in situ* reduction of HAuCl_4_ within MOF-functionalized waste PET substrates (Fig. 1a).

PET@UiO-66 was used as the initial substrate, which was immersed in HAuCl_4_ (1 mM aqueous solution) for 3 hours, reaching adsorption equilibrium (SI Note 1, Fig. S1). The use of UiO-66 as a porous scaffold facilitates homogeneous uptake of the Au precursor. The substrate was then air-dried without additional purification to retain the maximum of HAuCl_4_ within the MOF pores. Finally, laser treatment was performed using an engraving machine (K4 V2.5, 405 nm), resulting *in situ* reduction of Au NPs in the polymer matrix. Successful laser-induced reduction of Au NPs was confirmed by UV-Vis spectroscopy, where a plasmon resonance peak at 550 nm appeared in contrast to the initial PET@UiO-66 (Fig. 1b). Control experiments performed on hydrolyzed PET prior to UiO-66 growth showed that laser irradiation can also induce Au NPs formation in the absence of the MOF layer (SI Note 2, Fig. S2 and S3), as evidenced by weak and broadened plasmonic features at 560 and 650 nm. However, without the MOF layer the PET surface exhibited pronounced roughness and spatially non-uniform Au NPs distribution, resulting in poor plasmonic definition and limited control over laser patterning.

A homogeneous distribution of reduced Au NPs on the material is a key requirement for the microfluidic channel design, which depends on two factors: (i) the amount of chemisorbed Au precursor within UiO-66 pores and (ii) the laser treatment parameters. Precursor sorption capacity was preliminarily evaluated by monitoring HAuCl_4_ concentration over time, which remained unchanged after 3 hours of immersion. Therefore, optimization was focused on the laser treatment parameters, *i.e.*, the power and speed, using the Nelder-Mead method (SI Note 3, Table S1 and Fig. S4). The integrated area of the plasmon peak at 550 nm in the UV-Vis spectra was used as an optimization descriptor (Fig. 1c). The homogeneous distribution on the PET sheet (2 × 2 cm^2^) was achieved using the following laser parameters: 645 mW power and 6.2 mm s^−1^ speed (Fig. 1d). Additionally, the optical image of the material obtained under optimized conditions was analysed to evaluate the root mean square roughness (RMS). Since the color change (from white to pink) reflects the degree of NPs reduction, the RMS of pixel brightness was employed as a quantitative parameter at the macroscale (Fig. 1e). The obtained RMS value of 0.1 indicates low variations in color intensity and, consequently, a high degree of uniformity in the distribution of Au NPs inside the polymer matrix.

### Surface reconstruction and chemical transformation of PET@UiO-66 under laser-induced Au nanoparticle formation

**Fig. 2:**
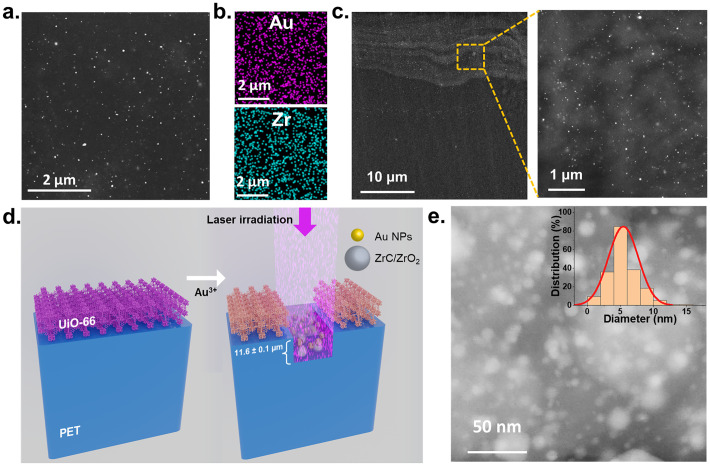
(a) SEM image of the PET@Au surface; (b) elemental maps of Au and Zr by EDX; (c) cross-sectional SEM image of PET@Au, showing Au NPs distribution within laser-modified layer with a penetration depth of 11.6 ± 0.1 µm; (d) schematic illustration of laser-induced *in situ* Au NPs reduction on the PET@UiO-66 surface; (e) STEM image with the corresponding Au NPs size distribution, revealing an average particle size of 5.4 ± 0.1 nm.

Homogeneous distribution of Au NPs and Zr was confirmed by scanning electron microscopy combined with energy-dispersive X-ray spectroscopy (SEM-EDX), as shown in Fig. 2a. The brightest regions indicate electron-rich Au, while the darker areas correspond to Zr-containing compounds such as UiO-66 and its treated forms (ZrO_2_), together revealing a homogeneous distribution of Au and Zr across the surface (Fig. 2b).

Before laser treatment, the UiO-66 coating exhibited a continuous layer with an average thickness of 600 nm (Fig. S5). After irradiation, cross-sectional SEM analysis revealed that the modified region extended to a depth of approximately 11.6 ± 0.1 µm (Fig. 2c), indicating that the laser locally melted both the MOF layer and the upper PET surface. We observe Au NPs penetration beyond the initial MOF thickness, suggesting that ZrO_2_ and incorporated Au diffused into the softened polymer matrix. Strong photothermal effects can explain this penetration during 405 nm laser irradiation.^23^ Localized heating at the MOF–PET interface causes partial melting of PET, while AuCl_4_^−^ ions are reduced to Au^0^ and redistributed within the molten polymer by thermally driven convection (Fig. 2d). The newly formed Au NPs enhance light absorption and local temperature, further extending the molten zone through plasmonic self-heating, resulting in an ≈11 µm modified layer. Similar nanoparticle embedding driven by laser-induced photothermal effects has been reported for polymer–metal systems, supporting this interpretation,^24,25^ and in our works.^21,26^ Zr-related transformations originating from decomposed UiO-66 were investigated further by X-ray photoelectron spectroscopy (XPS).

However, the individual Au nanoparticles could not be resolved in the cross-sectional SEM images due to their small size and embedding within the polymer phase. Therefore, to directly visualize the nanoparticles, the PET@Au composites were dissolved in hexafluoro-2-propanol, and the recovered Au NPs were analysed by scanning transmission electron microscopy (STEM) (Fig. 2e). The obtained nanoparticles exhibited an average diameter of 5.4 ± 0.1 nm, confirming their successful *in situ* formation during laser treatment. Although a small fraction of larger particles was present, their contribution to the number-weighted distribution was negligible, resulting in the observed narrow size profile in Fig. 2d.

**Fig. 3:**
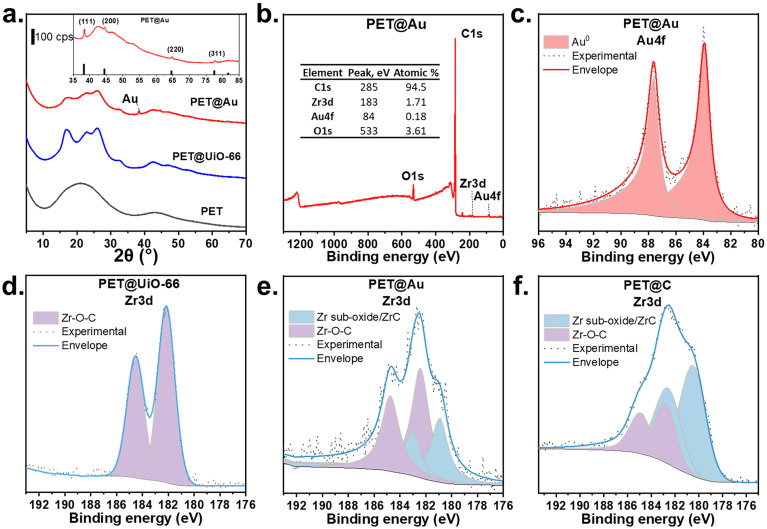
(a) XRD of initial PET, PET@UiO-66, and prepared PET@Au. The inset shows the PET@Au diffraction pattern alongside the reference peaks of metallic Au (JCPDS file: 04-0784); (b) XPS survey spectrum of PET@Au; (c) high-resolution spectrum of PET@Au in Au 4f region; (d) high-resolution spectrum of PET@UiO-66 in Zr 3d region, (e) high-resolution spectrum of PET@Au in Zr 3d region after depth profiling and (f) high-resolution spectrum of PET@C in Zr 3d region after depth profiling.

X-Ray powder diffraction (XRD) was used to confirm Au NPs formation within the composite after laser treatment (Fig. 3a). The initial PET and PET@UiO-66 showed the absence of the diffraction peaks corresponding to metallic Au. Diffractogram of PET@UiO-66 exhibited predominantly amorphous features of the MOF, in contrast to previously reported data.^21^ This behavior could be attributed to the reduced UiO-66 layer thickness and the initially low concentration of the precursors. After laser treatment, distinct diffraction peaks appeared at 38.3°, 44.5°, 64.7°, and 77.7° corresponding to the (111), (200), (220), and (311) planes of the metallic gold phase.^27–30^

Chemical composition of the PET@Au surface was confirmed by XPS. To probe the substrate region containing Au NPs embedded within the laser-modified PET@UiO-66 layer, the samples were etched using the monoatomic Ar^+^ depth-profiling technique (Fig. S6 and Table S2). Survey spectrum of PET@Au (Fig. 3b) revealed characteristic signals at 533 eV (O 1s), 285 eV (C 1s), 183 eV (Zr 3d), and 84 eV (Au 4f), confirming the presence of Au and Zr species.^31^ The high-resolution Au 4f spectrum (Fig. 3c) shows a typical doublet at 84 eV (Au 4f_7/2_) and 87.5 eV (Au 4f_5/2_),^32^ characteristic of metallic Au^0^, confirming Au NPs formation.

High-resolution Zr 3d spectrum of initial PET@UiO-66 (Fig. 3d, and Fig. S7, Table S3) shows a single doublet at 182.5 (184.8) eV corresponding to the Zr–O–C within the UiO-66 framework. After laser treatment of PET@UiO-66@Au^3+^ (Fig. 3e) and pristine PET@UiO-66 named as PET@C (Fig. 3f) additional doublet appeared at 180.8 (182.8) eV, assigned to Zr sub-oxides or ZrC species, indicating partial carbonization of UiO-66 during laser processing.^33^ According to Fig. 3c and e, no clear chemical interaction or alloy formation between Au and Zr was detected because the Au 4f and Zr 3d binding energies remain consistent with metallic Au^0^ and Zr–O/Zr–O–C environments, respectively. This suggests that Au NPs and the Zr-containing framework coexist as distinct phases. In the O 1s region intensity of the Zr–O signal at 529 eV decreases upon Au NPs formation (Fig. S8), further supporting partial structural modification of the UiO-66 framework during laser-induced reduction.

Quantification of the incorporated Au NPs is challenging because they are partially embedded in the PET. To isolate NPs, the PET@UiO-66 sheet (5 × 5 mm^2^) was dissolved in hexafluoroisopropanol, where the residual inorganic component was analyzed by inductively coupled plasma mass spectrometry (ICP-MS), confirming an Au content of 0.6 ng mm^−2^ in the PET sheet. Typically, 1 × 2 cm^2^ was the average size of the prepared PET@UiO-66 sheet, corresponding to only 30.5 ng of Au NPs presence. Our loading of 0.6 ng mm^−2^ corresponds to ∼3 pmol mm^−2^ and is sufficient to generate a dense plasmonic network while avoiding excessive aggregation.

### Plasmonic fields and optical response of embedded Au nanostructures

**Fig. 4:**
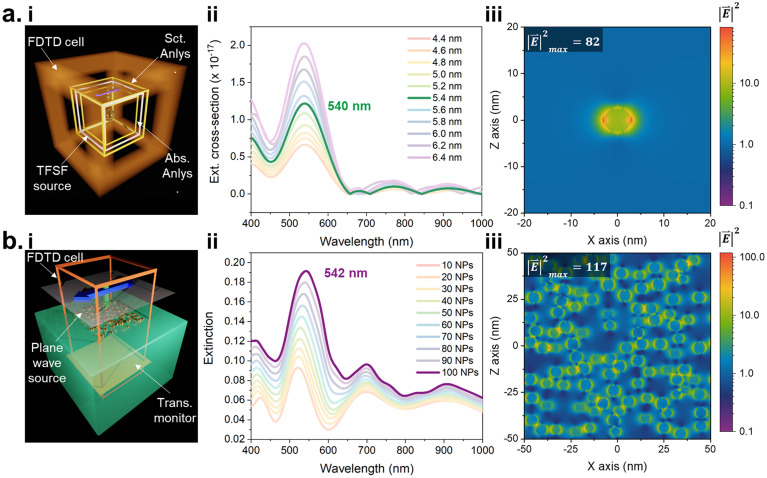
FDTD calculations for determining the plasmonic excitation conditions. (a) Single particle-based plasmonic nanoantennae: (i) simulation setup for calculating scattering and absorption cross-section from a single Au NP using a ‘Total field-scattered field (TFSF)’ source. (ii) Extinction cross-section as a function of Au NP diameter. (iii) Intensity profile in log scale showing enhancement due to excitation of antenna mode at 540 nm. (b) Plane-wave simulation of the random distribution of Au NPs in PET: (i) simulation setup for calculating transmittance, (ii) extinction as a function of Au NP number. (iii) Corresponding Intensity profile taken at the PET air interface showing plasmonic hot spots at 542 nm.

To elucidate how laser-induced Au nanoparticles interact with light inside the melted polymer matrix, we combined UV-Vis spectroscopy with finite-difference time-domain (FDTD) simulations (Fig. 4), revealing the plasmonic excitation behavior of Au NPs embedded in a PET matrix. The simulations were designed to capture both the single-particle optical response and the collective interactions that may arise in a random AuNP ensemble, providing an understanding consistent with the experimental observations. In the first model (Fig. 4a, i), a total-field scattered-field (TFSF) source was employed to investigate the response of an individual Au NP acting as a plasmonic nanoantenna,^5^ with the excitation polarized along the *x*-axis.

To mimic realistic conditions, the Au NPs size was systematically varied from 4.4 to 6.4 nm, reflecting the natural polydispersity observed experimentally in the laser-reduced PET@UiO-66 composites (Fig. 2e). The resulting extinction cross-sections (Fig. 4a, ii) reveal a distinct resonance centered around 540 nm, corresponding to the dipolar localized surface plasmon resonance (LSPR) mode of Au NPs in a dielectric medium (*n* = 1.575 for PET). As the diameter increases, the overall extinction intensity rises steadily, while the peak position remains nearly unchanged. Although the extinction cross-section is composed of both absorption and scattering contributions, the absorption term exceeds the scattering by roughly three orders of magnitude (Fig. S9). This confirms that, for such small particles, optical losses are dominated by absorption, leading to primarily non-radiative plasmon decay. The corresponding near-field intensity distribution (Fig. 4a, iii) clearly demonstrates the excitation of a dipolar nanoantenna mode, with an intensity enhancement maximum of 82 localized at the particle poles along the electric-field direction. The strong electromagnetic field confinement in the immediate vicinity of the Au NPs demonstrates the potential of these nanostructures to generate localized hot spots, which are crucial for photocatalytic reactions and plasmon-induced charge transfer processes.

To extend this analysis to more realistic conditions in which nanoparticles are randomly distributed within the PET matrix, a second simulation model^34^ was developed (Fig. 4b). Here, a plane-wave source illuminated the FDTD region containing multiple Au NPs dispersed at random positions with periodic boundary conditions to represent an infinitely extended PET@UiO-66 region. The calculated extinction spectra (Fig. 4b, ii) show a pronounced resonance at 542 nm, in excellent agreement with the single-particle LSPR (Fig. 4a, ii), confirming that the dominant optical response still arises from individual Au NPs. As the number of particles increases from 10 to 100, the LSPR mode becomes stronger due to collective resonances, a phenomenon well known in nanoparticle assemblies. The intensity map taken at the PET–air interface (Fig. 4b, iii) supports this interpretation; the field distribution shows pronounced heterogeneity with bright regions corresponding to coupled particle pairs or small clusters, yielding a maximum enhancement of 117. Since the Au NPs in the experimental PET@UiO-66 films are distributed at various depths within the polymer matrix, the extracted near-field pattern captures this realistic variation, with different particle sizes appearing at the *E*-field monitor due to these varied depths. Overall, our simulations validate the dual functionality of the laser-reduced Au NPs as efficient light absorbers and field enhancers, supporting their role as active components in plasmonic photocatalytic reactors.

Prior to plasmon catalytic experiments, we performed control measurement of PET@Au using 530 nm LED to exclude photothermal heating observed in case of laser-treated PET@UiO-66,^21^ which can arise in carbonaceous layer. To evaluate a potential photothermal contribution, the temperature of PET@Au was monitored during irradiation both in air and in aqueous media (Fig. S10a). While the surface temperature increased to approximately 54 °C in air after 1 h of irradiation, only a minor temperature rise (up to 30 °C) was observed when the material was immersed in water, indicating efficient heat dissipation under reaction conditions. Crucially, a control experiment conducted at 55 °C in the absence of PET@Au resulted in only 8% MB degradation after 50 min (Fig. S10b), which is negligible compared to the conversion achieved under plasmonic excitation at room temperature. These observations rule out bulk photothermal heating as potential contribution in further experiments.

**Fig. 5:**
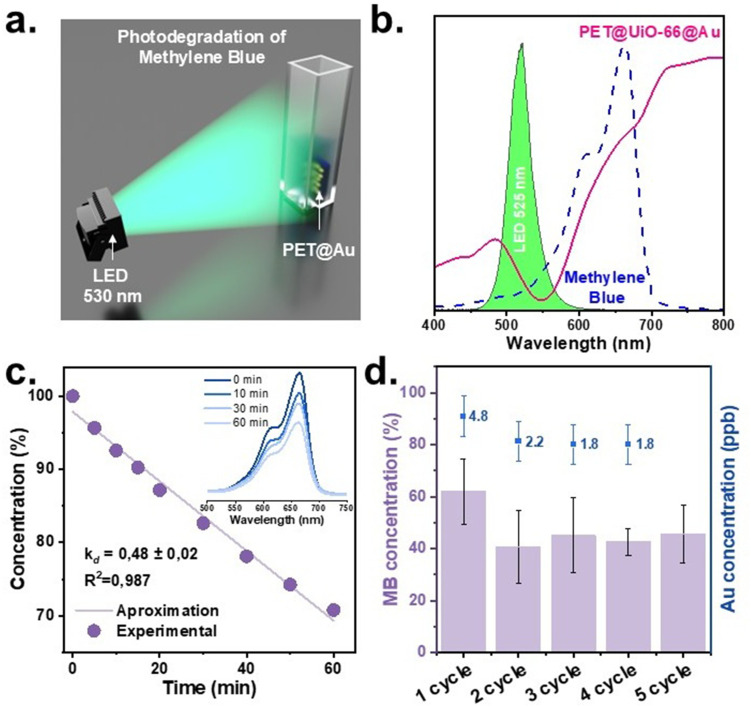
Catalytic activity of PET@Au evaluated using MB degradation as a model reaction. (a) Schematic representation of experiment setup; (b) optical spectra of PET@Au, initial MB solution, and LED emission centered at 530 nm; (c) kinetic curve of MB degradation using PET@Au with UV-Vis spectra recorded at 0, 10, 30, and 60 minutes; (d) results of reusability tests showing MB concentration after 5 consecutive reaction cycles using the same PET@Au and corresponding Au concentration in solution after each reaction as determined by ICP-MS (the data represents the mean value with standard deviation (SD) calculated from 3 experiments using 3 different material PET@Au (*N* = 3, *n* = 3)).

The plasmon activity of the PET@UiO-66 was evaluated through the degradation of methylene blue (MB), selected as a model reaction due to its strong absorption at 665 nm, which minimizes direct photolysis under irradiation (Fig. 5a). The PET@UiO-66 sheet (1 × 2 cm^2^) containing 1.5 × 10^−8^ mol of Au NPs was placed in an optical cuvette with MB aqueous solution (2.5 × 10^−5^ M, 3 mL) and was kept in the dark for 30 minutes to reach the adsorption–desorption equilibrium (Fig. S12). Then irradiation was performed using an LED at 530 nm, which excites the LSPR of PET@UiO-66 (550 nm). A schematic representation of the experimental setup is shown in Fig. 5b.

Despite an extremely low surface concentration of Au NPs (0.5 × 10^−11^ M), 33% of MB was degraded after 1 hour of irradiation, confirming the plasmon-assisted photocatalytic activity of the obtained PET@UiO-66. To further elucidate the origin of the observed activity, wavelength-dependent irradiation experiments^35^ were conducted using 530, 660 and 780 nm LEDs to independently excite either the plasmonic nanostructure or the dye molecule (SI Note 4, Fig. S11). Upon irradiation at 530 nm (overlapping with LSPR of PET@Au, but lying outside the absorption band of MB), a 33% degradation of MB was observed, indicating that direct molecular photolysis is not required for reaction initiation. In contrast, irradiation at 660 nm, matching the intrinsic absorption of MB, resulted in near-complete degradation (94%). Negligible conversion was detected at 780 nm, where neither the plasmonic substrate nor MB absorbs, excluding non-specific thermal or light-induced effects. Taken together, these results demonstrate that selective excitation of the plasmonic component alone is sufficient to drive catalytic MB degradation, confirming a plasmon-mediated reaction pathway under visible light irradiation.

Turnover number (TON = 132) and turnover frequency (TOF = 0.04 s^−1^) were calculated using Au concentration in PET@Au, assuming full accessibility of Au atoms. Even with this assumption, obtained values represent significant values compared to previous reported^36–39^ plasmonic and photocatalytic materials for MB degradation, as shown in Table 1. Moreover, in fact, TON and TOF values are sufficiently higher due to hindered access of MB to Au NPs incorporated or partially embedded in the polymer matrix. The apparent quantum yield (AQY) was found to be 1.56 × 10^−5^%, which is notably high considering the extremely low Au NPs loading. This value exceeds AQYs typically reported for the photodegradation of MB on plasmonic and photocatalytic materials (Table 1), indicating highly efficient photon utilization.

**Table 1:** Comparison of TON and TOF values reported for various photocatalytic and plasmon materials in the degradation of MB

Material	Irradiation source	Power	TON	TOF, s^−1^	AQY^a^, %	Ref.
TiO_2_	Xe arc lamp	150 W^c^	0.4 × 10^−3^	0.5 × 10^−7^	1.07 × 10^−1^0	36
TiO_2_(Nb : N)	Xe arc lamp	150 W^c^	9.7 × 10^−3^	1.3 × 10^−5^	2.48 × 10^−1^0	36
Ca-alginate supported ZnO NPs	Solar simulator light lamp	14.6 mW cm^−2^	5.43 × 10^−3^	1.5 × 10^−6^	1.93 × 10^−10^	37
Bi_2_SbVO_7_	Xe arc lamp	300 W^c^	1	1.54 × 10^−5^	3.93 × 10^−10^	38
Au-hydroxyapatite NPs	Xe lamp	10 W^c^	7.23	2.23 × 10^−4^	1.2 × 10^−11^	39
Ag NPs supported on diamond NPs	Solar simulator light lamp	86 ± 17 mW cm^−2^	476	0.05	5.15 × 10^−7^	40
Au NPs	Solar irradiation	0.137^b^	0.02	3.7 × 10^−6^	3.89 × 10^−6^	41
PET@Au	LED 530 nm	130.8 mW cm^−2^	132	0.04	1.56 × 10^−5^	This work

a AQY was calculated per 1 cm^2^.

b In the absence of reported data. Irradiance power of 340 W m^−2^ was assumed.

c In the absence of reported data. Irradiation area of 1 cm^2^ was assumed.

The reusability of the laser-induced PET@UiO-66 material as one of the key performance parameters in catalysis^42^ was evaluated over ten consecutive cycles of MB degradation under identical experimental conditions. The catalytic performance remained nearly unchanged throughout cycles, demonstrating the high stability of the immobilized Au NPs (Fig. 5d and Fig. S13). Residual MB concentration remains within the corresponding standard error of the earlier cycles, with no systematic loss of activity. To assess possible NPs leaching, in the first 5 cycles, each MB solution was collected after ICP-MS analysis of the reaction mixture. The concentration of dissolved Au was found to be 2.7 ± 2.1 ppb (Fig. 5d), which is close to the detection limit of the instrument and corresponds to trace amounts only. Taken together, these results confirm the stability and reusability of the PET@UiO-66, indicating that the laser-reduced Au NPs are firmly immobilized on the PET surface and do not undergo significant loss during repeated catalytic experiments.

### Towards laser-patterned microfluidic architectures for plasmonic flow catalysis

**Fig. 6:**
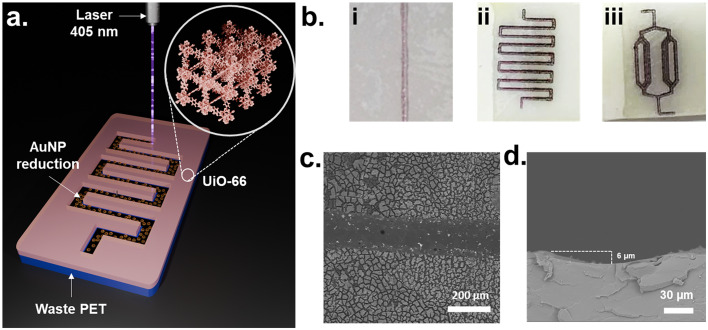
(a) Schematic showing the direct patterning of a microfluidic channel on the material. (b, i–iii) Photos of different branched channel schemes; (c) SEM image of a single channel, and (d) its cross-section.

The next step of our study focused on evaluating the potential of the proposed approach for one-step *in situ* Au NPs reduction combined with channel patterning. Fig. 6a schematically illustrates the process, where laser patterning induces the formation of channels with reduced Au NPs. Straight channel and several channels with more developed structural patterns were fabricated as a proof of concept (Fig. 6b). Their width was measured to be 160 ± 13 µm by SEM (Fig. 6c), while the maximum channel depth determined by cross-section SEM reached approximately 6 µm (Fig. 6d). Confocal surface profilometry (Fig. S14) revealed an apparent depth of about 15 µm and provided a macroscale view of the channel topography, confirming the continuous and well-defined structure of the laser-treated region. The higher depth value observed by confocal microscopy is attributed to optical contributions from surface roughness and refractive index variations within the reduced Au in the PET zone.

Subsequently, this method was tested to fabricate various complex channel geometries resembling microfluidic chip architectures.^8^Fig. 6a and b show three representative designs obtained on the PET@UiO-66. It should be noted that the total irradiation time required to pattern a 1 × 2 cm^2^ area didn't exceed 40 minutes. This approach significantly simplifies the fabrication of plasmon-active microfluidic chips for catalytic applications by combining material synthesis and channel patterning into a single laser-assisted step. Considering the negligible Au concentration and use of low-cost PET substrate, PET@Au represents a promising and economically viable platform for plasmon-assisted microfluidic applications.

## Conclusion

In conclusion, we developed a conceptually new route for the design of a plasmon-active microfluidic system through a laser-assisted fabrication strategy that combines *in situ* Au NPs formation with controlled channel patterning. The laser treatment performs two functions at once that induce the Au NPs reduction and simultaneously create structured channels on the surface. Usage of MOF-functionalized waste PET substrate allowed for uniform distribution of the Au precursor across a 1 × 2 cm^2^ surface. As a result, Au NPs (≈5.4 nm) absorbing at 550 nm were homogeneously reduced and embedded throughout the material. FDTD simulations correlated the experimentally observed plasmon resonance at ≈550 nm with strong electromagnetic field localization around the embedded Au NPs, confirming efficient light–matter coupling responsible for the observed photocatalytic activity. The obtained plasmon-active PET@Au composite demonstrated photocatalytic performance in the MB degradation. The TON reached 132 and TOF was 0.04 s^−1^, which are higher than many previously reported photocatalytic systems. Moreover, Au NPs were incorporated into the polymer matrix to a depth of 11.6 ± 0.1 μm, which prevented their leaching and eliminated the need for additional purification steps after reaction. The fabricated microchannels exhibited a width of 160 µm and a channel depth of 6 µm. The patterning parameters can be easily adjusted to obtain complex microfluidic architectures for further studied in plasmonic microfluidic applications. Such features open the way toward compact, recyclable, and energy-efficient light-driven microreactors capable of pollutant degradation, selective photocatalysis, or *in situ* spectroscopic monitoring.

Furthermore, this work builds on our previous studies on the functional upcycling of waste PET *via* covalent attachment to a MOF *via* terephthalic acid (in both PET and the MOF).^43^ It was shown that the surface of PET-functionalized by UiO-66 ^21^ and Ni-BDC^26^ able for control laser patterning. Crucially, this approach is generalizable. By employing different MOFs with terephthalic acid as the organic linker (Ni-BDC, MIL-53(Al), MOF-5 (Zn) and Cu-BTC), it is possible to systematically vary the pore architecture and surface chemistry of the functional layer. This, in turn, provides powerful handle to control nucleation environment during *in situ* laser-assisted reduction, allowing in perspective tuning of the resulting PNPs morphology, size and distribution.

Thus, our method addresses several key challenges in plasmonic catalysis. It reduces the cost of materials by minimizing the amount of noble metal required, simplifies the fabrication process by replacing multistep procedures with a single laser-assisted step, and ensures the stability of the material within the reaction solution. At the same time, it enables direct microchannel formation, creating a foundation for scalable plasmonic flow reactors that combine light harvesting, catalytic functionality, and sustainable materials design in one architecture.

## Materials and methods

### Materials

Transparent recycled polyethylene terephthalate (PET) was purchased from hardware stores in Tomsk city (Russia). All chemicals used were of analytical grade or of the highest purity available to us. HAuCl_4_·3H_2_O (≥99.9%), ethanol, methanol, *N*,*N*-dimethylformamide (reagent grade, ≥99%), methylene blue hydrate (≥97.0%), ZrCl_4_ (≥99.9%), H_2_SO_4_ (≥95%), HCl (37%) were purchased from Sigma-Aldrich and Alfa used without further purification. All experiments were performed in deionized water. All glassware was thoroughly cleaned with freshly prepared 3 : 1 HCl/HNO_3_ (aqua regia) and rinsed thoroughly with water prior to use.

### PET@UiO-66 preparation

PET sheets were functionalized with UiO-66 using a modified procedure based on a previously reported method.^21^ The process consisted of two main steps: surface hydrolysis and UiO-66 growth.

#### Surface hydrolysis

PET sheets were cut into 2 × 2.5 cm^2^ rectangles, and a 2 × 2 cm^2^ hydrolysis zone was defined on each sample. A protective polyethylene film was removed from this area, and the exposed PET surface was placed on top of the concentrated sulfuric acid at 80 °C for 5 minutes in a Petri dish. After the required time, the surface-hydrolyzed PET sheet was removed and washed with distilled water. It was then subjected to ultrasonic treatment and dried in air overnight.

#### UiO-66 growth

The pretreated PET was then placed in a Teflon-lined autoclave, where ZrCl_4_ (31 mg), DMF (10 mL), and concentrated HCl (0.25 mL) were added. The mixture was stirred for 10 min before adding terephthalic acid (31 mg) as an organic linker. The autoclave was maintained at 80 °C for 15 h and then allowed to cool naturally to room temperature. The resulting PET@UiO-66 was washed sequentially with DMF and methanol to remove unreacted species and loosely bound crystals, followed by vacuum activation at 50 °C overnight.

### Preparation of PET@Au

PET@UiO-66 sheets were immersed in HAuCl_4_ (1 mM, 10 mL) for 3 hours to allow adsorption of Au^3+^ ions into the UiO-66 layer. After immersion, the samples were dried in the air overnight without further purification. Laser-induced reduction of Au^3+^ to Au NPs was performed using a pulsed diode laser NEJE DK-8-KZ at a wavelength of 405 nm, pulse frequency of 1.6 kHz, and a rated power of 1500 mW. The laser beam was focused on the material surface, and irradiation was applied across the entire active area of the sheet as the laser head scanned along predefined trajectories. The average laser power was adjusted by controlling the pulse duration and frequency in the laser control system.

Optimization of the laser-induced reduction parameters was carried out using the Nelder–Mead method (SI Note 3), in which laser power and scanning speed were varied. The area of the plasmon resonance peak was obtained by numerical integration over an individually selected wavelength window around the plasmon resonance center (typically 480–650 nm) and used as the objective function in the Nelder–Mead optimization. The optimal conditions for achieving homogeneous distribution of Au NPs were identified as 645 mW laser power and 6.2 mm s^−1^ scanning speed.

### Catalytic activity tests

Degradation of MB was used to evaluate the catalytic activity of the PET@Au. PET@Au (1 × 2 cm^2^) was placed in the MB aqueous solution (3 mL, 2 × 10^−5^ M) in a quartz cuvette. Prior to irradiation, the sample was kept in the dark for 30 minutes to reach adsorption–desorption equilibrium between the dye and the catalyst surface. Reaction was carried out under illumination with an LED source (530 nm) for up to 60 minutes. After irradiation, PET@Au was removed, and the residual MB concentration was analyzed using UV-Vis spectroscopy. For reusability tests, reaction was carried out 3 times to estimate the standard deviation according to the following equation: 
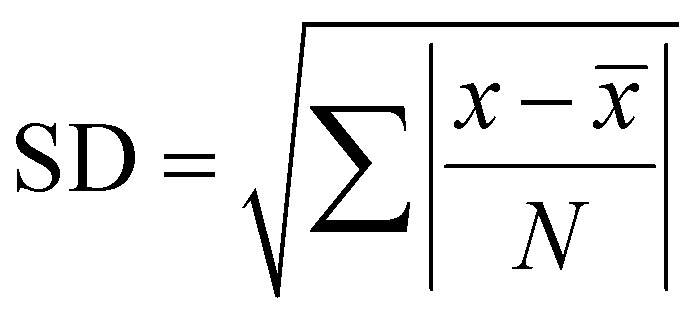
, where *x* is the value of residual MB concentration, 
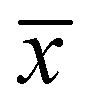
 is the mean of residual MB concentration and *N* = 3. After each cycle the PET@Au sheet was sequentially washed with ethanol 3 times (10 mL, 20 min each) and then with DI water, followed by drying in air before reuse in the next cycle. Temperature of material was measured using thermal imaging camera HT-02 (HTI, China). Camera was fixed on the material and during irradiation temperature was fixed at 0, 10, 20, 30, 40, 50 and 60 minutes of irradiation by LED 530 nm.

### Turnover number and frequency calculation

Turnover numbers (TON) were calculated as the ratio between the amount of product formed and the amount of catalytic active centers, according to:
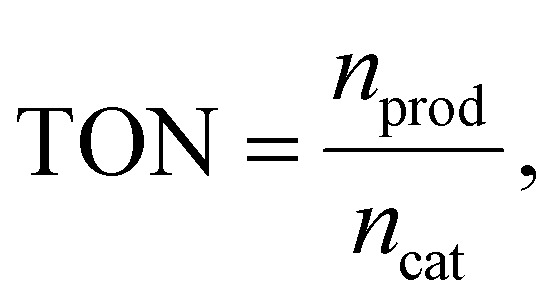
where *n*_prod_ is the amount of degraded MB (mol); *n*_cat_ is the amount of catalyst (mol).

For PET@Au *n*_cat_ corresponds to the total amount of Au (mol) determined by ICP-MS, used as an upper-limit estimate of the accessible catalytic sites.

Turnover frequencies (TOF) were calculated according to:
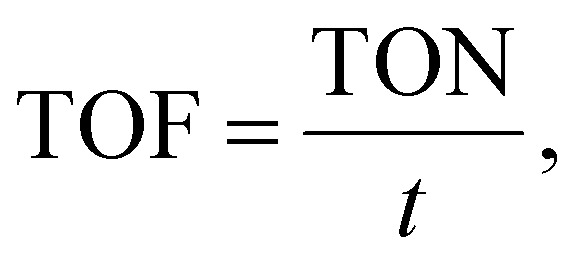
where *t* is reaction time (s).

### Apparent quantum yield calculation

Apparent quantum yields (AQY) were calculated using
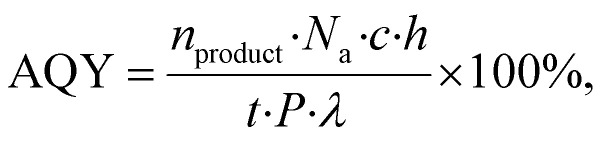
where *N*_a_ – the Avogadro number (6.022 × 10^23^ mol^−1^); *h* – Planck's constant (1.054 × 10^−34^ J s); *c* – the speed of light (2.998 10^8^ m s^−1^); *t* – the illumination time (s); *λ* – the emission wavelength (m); and *P* is the radiant power (W).

For broadband Xe lamp and solar simulator, the energy of incident photon flux was calculated by integrating the spectral irradiance over the corresponding wavelength ranges (400–700 nm for Xe and 350–1000 nm for solar). The AQY was then calculated as:
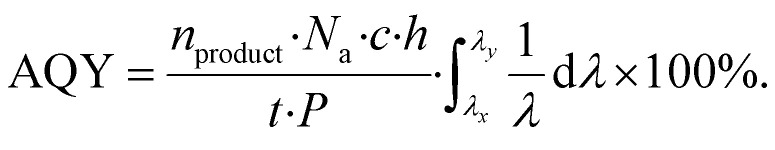


The main aim was to compare the photocatalytic performance with literature data. AQY was determined using a standard irradiated area of 1 cm^2^.

### Characterization methods

#### UV-Vis spectroscopy

Adsorption spectra of methylene blue and HAuCl_4_ aqueous solutions were recorded using the Analytik Jena SPECORD250+ spectrometer in the 350–1100 nm range with 200 nm min^−1^ scanning speed and spectral resolution of 1 nm. Deionized water was used as a reference blank. Diffuse reflectance spectra of the solid samples were recorded on the same instrument equipped with an integrating sphere module. Polytetrafluoroethylene was used as a reflectance standard.

### X-Ray photoelectron spectroscopy

XPS spectra were recorded on a Thermo Fisher Scientific XPS NEXSA spectrometer equipped with an Al Kα X-ray monochromatic source at 1486.6 eV. Survey spectra were acquired with a pass energy of 200 eV and an energy resolution of 1 eV. High-resolution spectra (C 1s, N 1s, O 1s, S 2p, Au 4f) were collected using a pass energy of 50 eV and a resolution of 0.1 eV. The spectra were calibrated against the C 1s peak set at 284.8 eV. Elemental concentrations were determined in at% using the instrument sensitivity factors supplied by the manufacturer. The analysis area was 200 µm^2^. A flood gun was used to compensate for the charges. A monoatomic gun was used for depth profiling with an energy of 4000 eV. High-resolution spectra were fitted using a mixed Gaussian–Lorentzian line shape with Shirley background subtraction.

### Scanning electron microscopy with energy dispersive X-ray analysis (SEM-EDX)

The images were taken on a Zeiss Merlin microscope operated in the secondary electron and backscattered electron modes. The instrument was equipped with an Oxford Instruments INCAx-Act Energy Dispersive X-Ray detector. Prior to measurements, all samples were coated with a thin carbon layer by magnetron sputtering to prevent charging. SEM observations were performed at an accelerating voltage of 10 kV. For cross-sectional analysis, samples were frozen in liquid nitrogen and fractured to expose the internal structure.

### Scanning transmission electron microscopy (STEM)

Annular dark field STEM imaging of the Au NPs was performed using a FEI/Thermo Fisher Scientific TECNAI G2 F20 field-emission (scanning) transmission electron microscope operating at 200 kV. Before analysis, PET@Au was dissolved in hexafluoro-2-propanol (HFIP) to release embedded Au NPs. The dispersion was centrifuged to isolate Au NPs, and the supernatant solvent was subsequently replaced with methanol.

### Optical and confocal microscopy

Optical images were obtained using a Leica S9i microscope. 3D surface topography of the material was analysed using a µsurf explorer confocal microscope (NanoFocus AG, Germany).

### ICP-MS

Elemental dissolution was assessed using Inductively Coupled Plasma Mass Spectrometry (ICP-MS, Agilent 7900, Agilent Technologies), equipped with a collision cell operating with helium as the cell gas at a flow rate of 5 mL min^−1^. Prior to analysis, the instrument was calibrated using a standard solution containing 50 mM of the working solution prepared in ultrapure water. After dissolving PET in hexafluoro-2-propanol, the released Au NPs were dissolved in aqua regia (HNO_3_/HCl) and subsequently diluted with ultrapure water. The analyte solution was delivered at a controlled rate of approximately 5.6 ± 0.4 mg s^−1^ using pressurized argon. Before nebulization and introduction into the plasma torch, the analyte was mixed with a standard solution, with yttrium used as the internal standard. Each sample was analyzed for over 15 minutes, and the resulting counts per second were converted to ng s^−1^ using the calibration curve.

### X-Ray powder diffraction (XRD)

The analysis of the crystallographic phases was performed based on X-ray powder diffraction experiments in Bragg Brentano geometry with a scattering angle range of 3° < 2*θ* < 85° measured on a Malvern PANalytical (B.V.) Empyrean diffractometer. A focusing mirror was used to provide Cu Kα_1,2_-radiation for the experiment. The beam divergency was defined by using a 1/4° fixed vertical entrance slit, followed by a 0.04 rad horizontal Soller slit and a 0.04 rad horizontal Soller slit on the secondary side in front of an open 2D GaliPix detector. Detector to sample distance for this instrument was fixed to 240 mm. Instrument calibration for both line position and instrumental broadening was performed using NIST Standard Reference Material® 660a (Lanthanum Hexaboride).

### Finite-difference time-domain (FDTD) simulation

Finite-difference time-domain (FDTD) simulations were performed using a commercial 3D electromagnetic solver by Ansys Lumerical^44^ to study the optical extinction of Au NPs embedded in PET medium. An automatic non-uniform mesh with a minimum step of 0.25 nm and an auto-shutoff of 1 × 10^−5^ was applied. In the first model, a total-field scattered-field (TFSF) source spanning 400–1000 nm was used to compute the extinction cross-section of a single spherical AuNP (diameter = 5.4 nm, varied for size-dependent analysis) embedded in PET (*n* = 1.575). Perfectly matched layer (PML) boundaries were used in all directions, with *x*-polarized excitation and absorption/scattering calculated from power flow inside and outside the TFSF region. The source, absorption, and scattering boxes were 100 × 100 × 100 nm^3^, 80 × 80 × 80 nm^3^, and 120 × 120 × 120 nm^3^, respectively, with a 1 nm mesh throughout and a 0.1 nm mesh locally around the particle. In the second model, a plane-wave source was applied to a 100 × 1400 × 100 nm^3^ FDTD region, containing 100 randomly distributed Au NPs on a PET substrate. Periodic boundaries were set along *X* and *Z*, with PML along *Y*. Transmittance (*T*) was recorded within PET, and extinction was calculated as Ext = −ln *T*. A 0.5 nm mesh covered the particle region (100 × 20 × 100 nm^3^), and the electric field at the PET–air interface was extracted at the localized surface plasmon resonance (LSPR) wavelength. The dielectric function of gold was taken from Johnson and Christy,^45^ whereas the PET substrate was modeled after Zhang *et al.*^46^

## Author contributions

A. G.: investigation, writing – original draft, data curation, methodology, validation; S. S.: electromagnetic simulations, visualization, writing – original draft (simulation section), writing – review & editing; D. K.: investigation, methodology; W. F. V.: methodology; M. O.: data curation, validation; T. S.: methodology; H. K.: investigation; A. I.: investigation, methodology; M. V.: resources; P. P.: conceptualization, writing – review & editing, supervision, funding acquisition; O. G.: conceptualization, supervision, supervision, writing – original draft.

## Conflicts of interest

There are no conflicts to declare.

## Supplementary Material

NR-018-D5NR05068E-s001

## Data Availability

The data supporting this article have been included as part of the supplementary information (SI). Supplementary information: additional experimental details, supporting figures (SEM, UV-Vis, optical and confocal images, XPS), tables, and notes. See DOI: https://doi.org/10.1039/d5nr05068e.
