# Supplementary material for: Laser writing of plasmonic catalytic microchannels on UiO-66 layer
Source: Nanoscale. 2026 Feb 23;18(12):6369–80. doi: 10.1039/d5nr05068e (PMC12934202; doi:10.1039/d5nr05068e)
Supplement: NR-018-D5NR05068E-s001 [file NR-018-D5NR05068E-s001.pdf]

Supplementary Information (SI) for Nanoscale.
This journal is © The Royal Society of Chemistry 2026

Supporting information for

Laser Writing of Plasmonic Catalytic Microchannels on UiO-66 layer

Alina Gorbunova[1,2], Swagato Sarkar[3,4], Dmitry Kogolev[1], Wanderson Ferraz do Valle[2], Markus Ostermann[2], Thomas Schachinger[5], Hradil, Klaudia[6], Alexey Ivanov[1], Markus Valtiner[2], Pavel Postnikov[1,7*], Olga Guselnikova[2*]

1. *Research School of Chemistry & Applied Biomedical Technology, Tomsk Polytechnic University, Tomsk, Russia*
2. *Institute of Applied Physics, Technical University of Vienna, Vienna, Austria*
3. *Leibniz-Institut für Polymerforschung Dresden e.V. (IPF), Institute for Physical Chemistry and Polymer Physics, Hohe Str. 6, 01069 Dresden, Germany*
4. *Life Science Nanomikrosysteme, Helmholtz-Zentrum Dresden-Rossendorf, Bautzner Landstraße 400 – 01328 Dresden, Germany*
5. *University Service Centre for Transmission Electron Microscopy, TU Wien, Vienna, Austria*
6. *X-Ray Center, TU Wien, Vienna, Austria*
7. *N.N. Vorozhtsov Institute of Organic Chemistry, Pr. Ak. Lavrentieva 9, Novosibirsk 630090, Russia*

*Corresponding authors: Olga Guselnikova, Pavel Postnikov

Email: olga.guselnikova@tuwien.ac.at, postnikov@tpu.ru

Table of Contents

Supplementary Note №1. PET@UiO-66 sorption capacity test 3

Supplementary Note №2. Laser-assisted reduction of the Au NPs on the hydrolyzed PET surface 4

Supplementary Note №3. Nelder-Mead optimization procedure...5

Supplementary Note №4. Control experiments of photothermal and plasmon effect evaluation 12

Supplementary Note №1. PET@UiO-66 sorption capacity test

PET@UiO-66 was immersed in 3 mL of the $HAuCl_4$ aqueous solution (1 mM) at room temperature for up to 6 hours. The sorption profile (Figure S3) demonstrates that the maximum uptake of HAuCl4 (14 %) was reached within 2 hours, after which the concentration remained nearly constant. Therefore, an immersion time of 3 hours was selected to ensure the complete saturation of the MOF pores with HAuCl4 prior to further optimization.

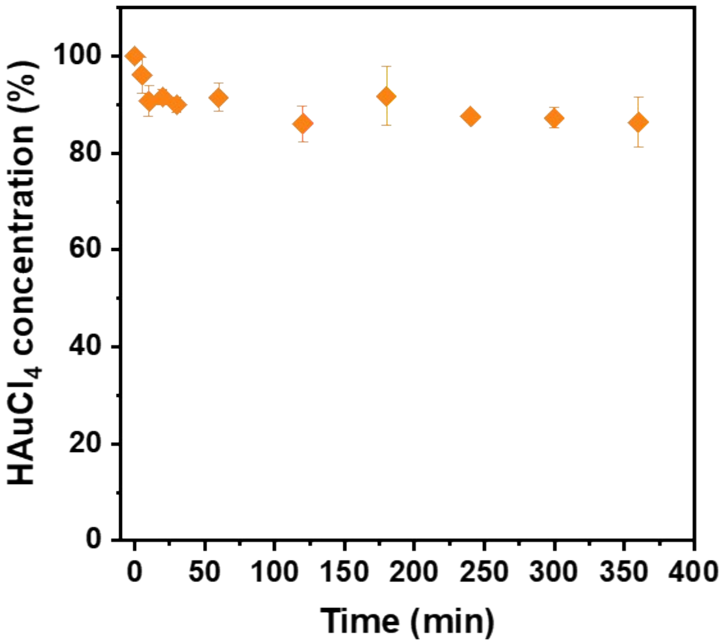

Figure S1. Kinetic plot of $HAuCl_4$(1 mM) sorption on PET@UiO-66, used to determine the maximum sorption capacity of the initial PET@UiO-66 and the maximum distribution of Au^{3+} within the MOF pores.

Supplementary Note №2. Laser-assisted reduction of the Au NPs on the hydrolyzed PET surface

Hydrolyzed PET (PET-H) was immersed in the aqueous $HAuCl_4$ for 3 hours. After drying, PET-H was subjected to laser treatment using parameters 645 mW power and 6.2 mm/s scanning speed. Following the laser irradiation, several pink-colored regions were observed on the PET surface, predominantly located near corners (Figure S2). The average UV-Vis spectrum (obtained from spectra corresponding to each quarter of the initial PET sheet) exhibited a weak reflectance peak at 560 and 650 nm (Figure S3). The rough surface of PET-H can physically adsorb some precursor, but MOF absence results in non-homogeneous distribution of reduced Au NPs on the area 2x2 cm^2 and consequently weak LSPR signal. Contrary, MOF layer in PET@UiO-66 accumulates $HAuCl_4$ inside pores, leading to homogeneous distribution of Au NPs, evidenced by strong LSPR peak at 550 nm.

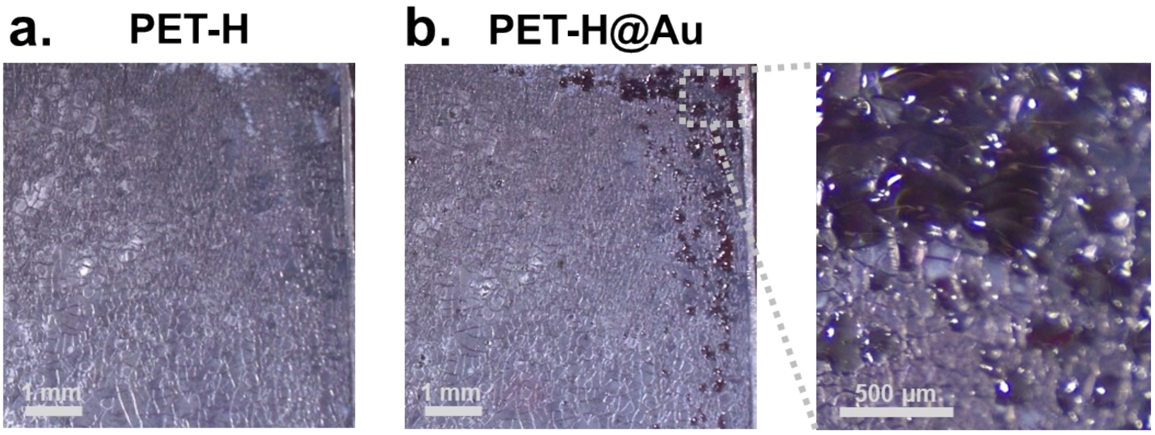

Figure S2. Optical image of a) hydrolyzed PET (PET-H) and b) laser-reduced Au NPs on PET-H (PET-H@Au)

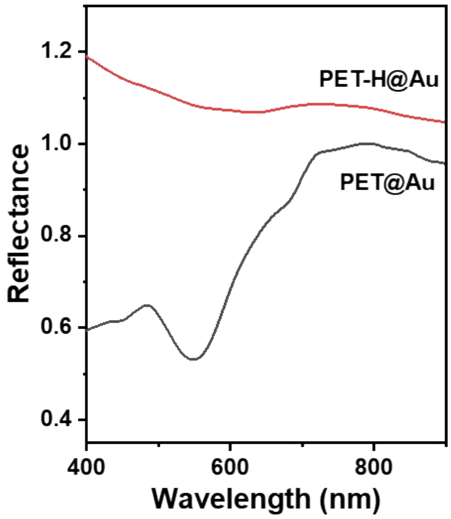

Figure S3. UV-Vis spectra of PET-H@Au and PET@Au

Supplementary Note №3. Nelder-Mead optimization procedure

Optimization of the laser processing parameters was performed using the Nelder-Mead algorithm, which enables multivariable optimization without gradient calculation.[1] A simplex consisting of n+1, where n is the number of variables, was initially constructed. The objective function was evaluated at each vertex, and the vertex corresponding to the worst function value was reflected relative to the centroid of the remaining vertices to form a new simplex. Depending on the evolution of the function values, reflection, expansion, contraction, or shrinkage operations were applied iteratively.

The optimization was continued until one of the following termination criteria was satisfied:

1) The target value of the objective function was achieved.
2) n+1 successive simplexes were generated without improvement in the objective function.

The optimized parameters were laser power (mW) and laser beam speed (mm/s). The objective of the optimization was to maximize the area of the plasmon resonance peak at 550 nm according to the UV-Vis reflectance spectrum of the sample. The highest area value (613 a.u.) corresponds to the optimal conditions: laser power 645 mW and laser speed 6.2 $mm \cdot s^{-1}$.

Table S1. Table of parameters for Nelder-Mead optimization

**Point**	**Laser power, mW**	**Laser speed, $mm \cdot s^{-1}$**	**Area**
**1**	645	6.2	613
**2**	645	8.9	76
**3**	750	6.2	326
**4**	750	4.7	557
**5**	645	4.7	346
**6**	720	5.8	384
**7**	675	4.9	500

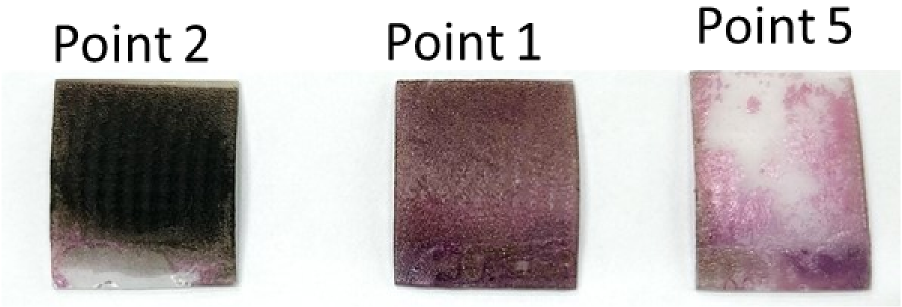

Figure S4. Optical images of the PET@Au at points 1, 2, and 3, respectively, during the Nelder-Mead optimization process of laser treatment parameters.

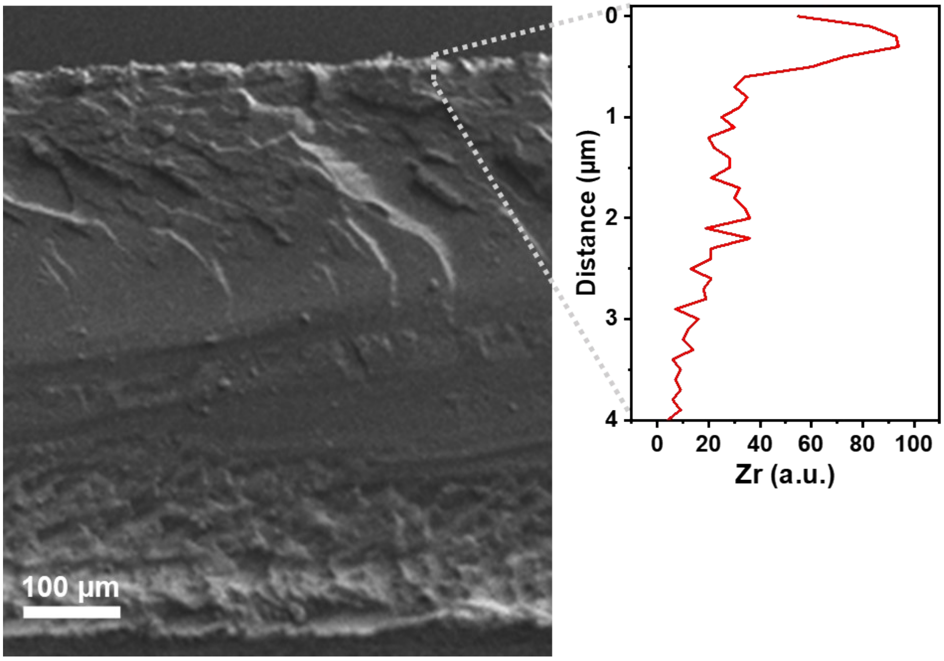

Figure S5. Cross-sectional SEM image of PET@UiO-66 combined with corresponding line EDX profile, showing the distribution of Zr across the material. The Zr signal extends approximately 600 nm.

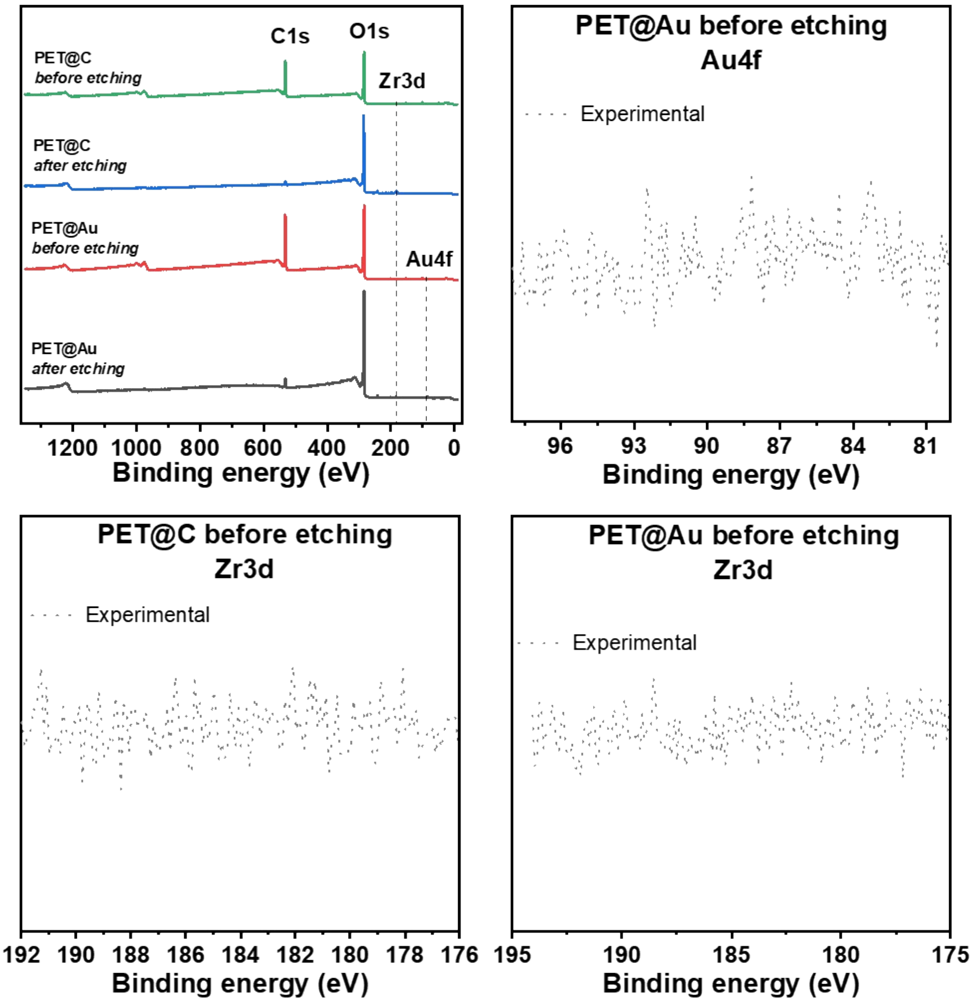

Figure S6. a) Survey spectra of PET@C and PET@Au before and after etching showing appearance of Zr3d and Au4f peaks after surface etching; b) High resolution spectrum of the Au4f region for PET@Au before etching; c) Zr3d for PET@C before etching; d) Zr3d for PET@Au before etching.

Table S2. Elemental composition (at.%) of the sample's surface determined by XPS survey spectra.

Sample	**C1s**		**O1s**		**Zr3d**		**Au4f**	
Etching	**before**	**after**	**before**	**after**	**before**	**after**	**before**	**after**
**PET@C**	77.34	96.03	22.66	3.34	0	0.64	0	0
**PET@Au**	75.41	65.59	24.59	4.21	0	0.14	0	0.07

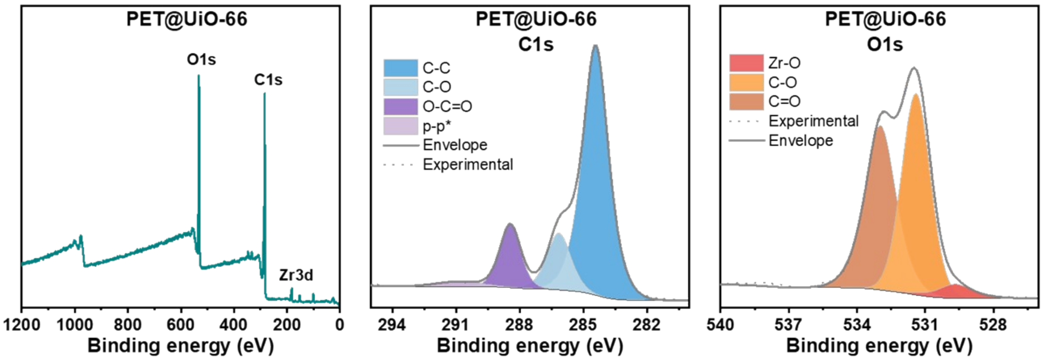

Figure S7. XPS spectra of initial PET@UiO-66: a) survey spectrum; b) C1s region; c) O1s region.

Table S3. Deconvolution parameters of high-resolution XPS spectra in the C1s and O1s regions for the initial PET@UiO-66 sample.

Name	Peak position, eV	Area, cps	FWHM fit param (eV)
C-C	284.81	67619.84	1.47
C-O	286.53	13900.12	1.39
O-C=O	288.84	13510.84	1.22
p-p*	291.26	2320.73	2.61
Zr-O	529.66	4109.43	1.78
C-O	532.95	43180.16	1.68
C=O	531.39	39613.17	1.32

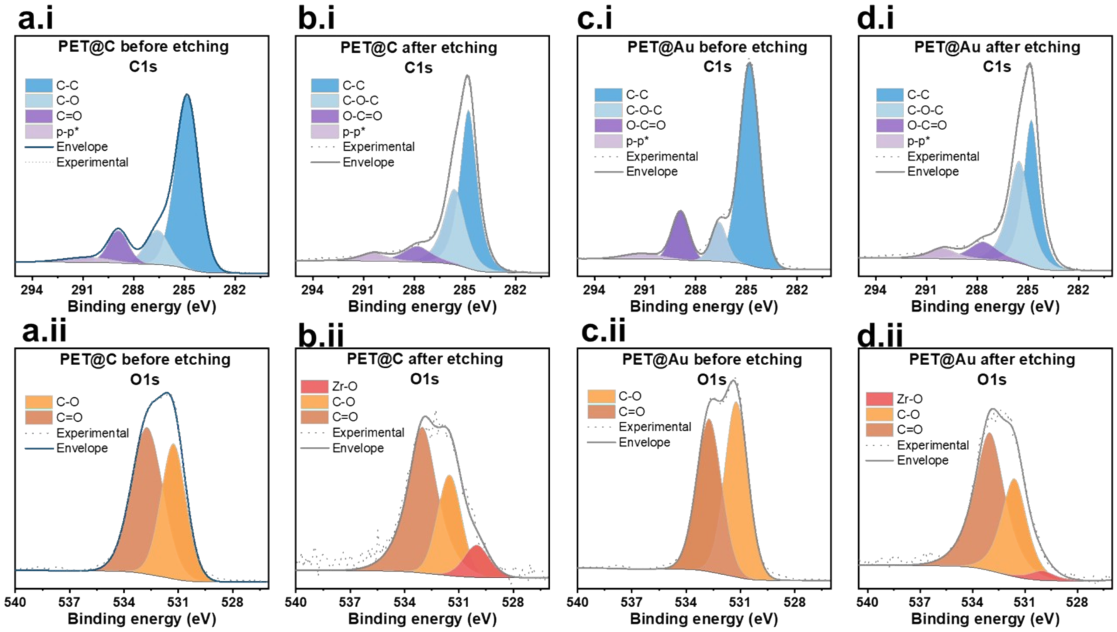

Figure S8. High-resolution spectra of a) PET@C before etching, b) PET@C after etching, c) PET@Au before etching, d) PET@Au after etching in i) C1s and ii) O1s regions

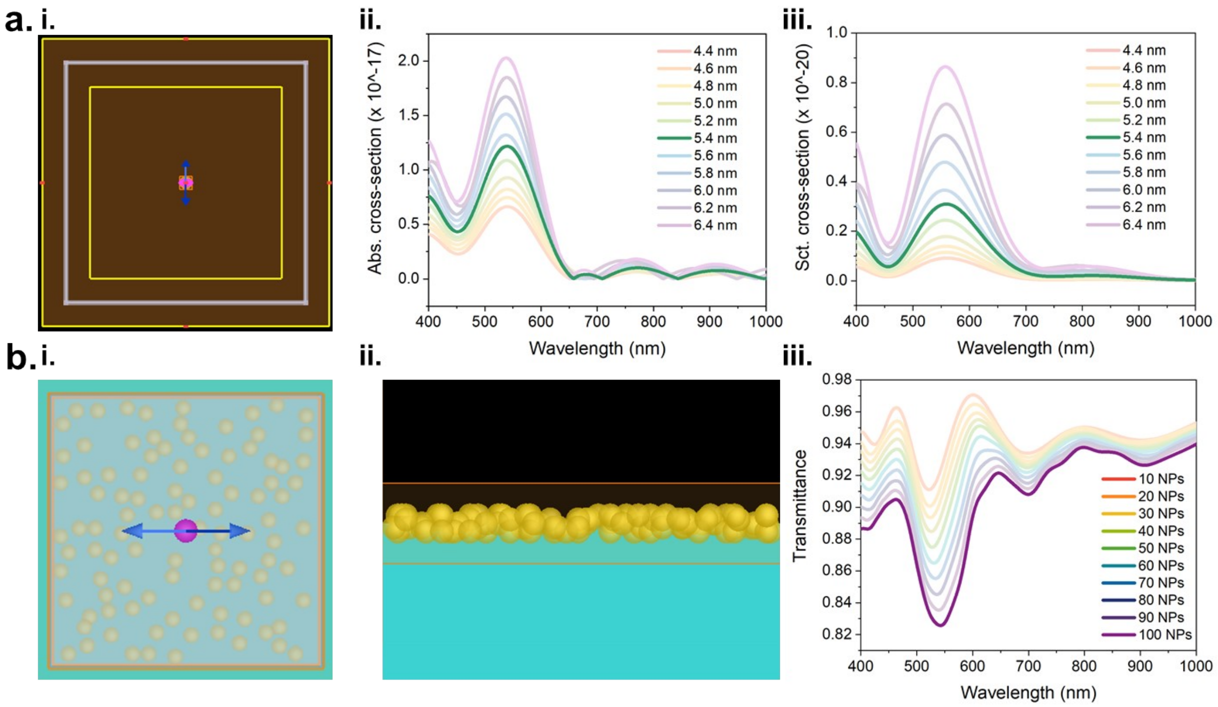

Figure S9. a) Single particle-based plasmonic nanoantennae: (i) Screenshot from the simulation software showing the single AuNP with TFSF source (white box) and absorption/ scattering analysis (yellow boxes). (ii, iii) Absorption and scattering cross section as a function of AuNP diameter. b) Plane wave simulation of the random distribution of AuNPs in PET: (i, ii) Top view and cross-sectional view of the PET substrate with 100 AuNPs arranged randomly. (iii) Transmittance as a function of the number of AuNPs.

Supplementary Note №4. Control experiments of photothermal and plasmon effect evaluation

To assess photothermal contribution, the temperature of PET@Au was measured during LED 530 nm irradiation in both water and air (Figure S10a). While the temperature of material at air reached up to 54 °C after one hour, temperature increase in H_2O (up to 30 °C) was negligible due to efficient heat dissipation. Moreover, control experiment of MB degradation at 55 °C without PET@Au resulted only 8 % degraded MB after 50 minutes (Figure S10b). This minor degradation is negligible compare to plasmon-initiated by PET@Au at r.t., confirming that observed reaction initiation is not driven by photothermal effect.

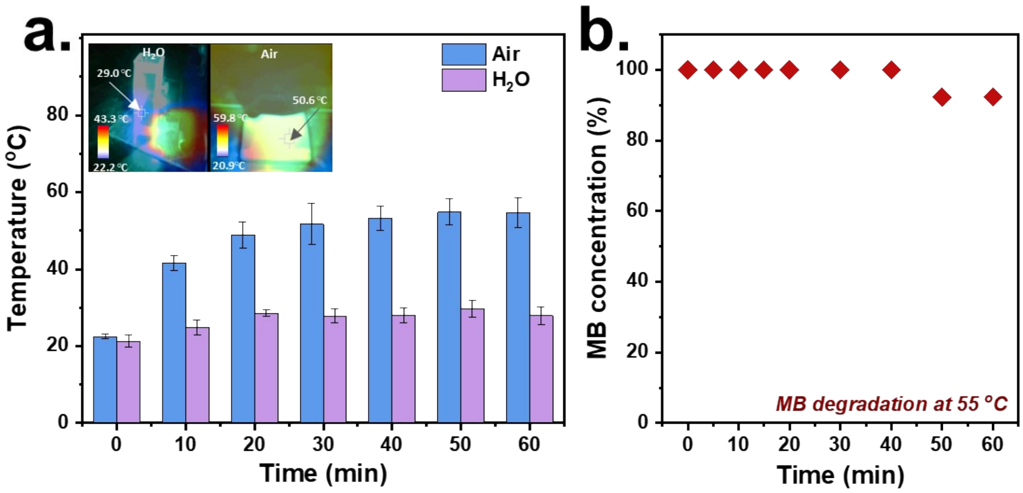

Figure S10. a) Temperature of material under irradiation at air and inside water. Thermal images of PET@Au in H_2O and at air under irradiation (30 minutes) are presented in the right corner. b) Blank experiment of MB degradation at 55 °C without PET@Au.

To evolute plasmonic contribution, we performed MB degradation under irradiation at different wavelengths (530, 660 and 780 nm). Selection was based on spectral overlap: 530 nm for direct plasmon resonance excitation of PET@Au (550 nm), 660 nm for direct excitation of MB molecule (665 nm) and 780 nm as a non-absorbing control for both (Figure S11). Intriguingly, the highest degradation efficiency (94 %) was observed under 660 nm irradiation. This suggests synergetic mechanism where direct MB excitation could be strongly enhanced by weak LSPR of PET@Au at this wavelength. In comparison, irradiation at the LSPR excitation (530 nm) resulted in 33% efficiency, while the control wavelength (780 nm) showed minimal activity (6 %). This indicates that plasmon excitation is active, however the most efficient pathway can be reached by coupling plasmonic effect and direct molecule excitation with plasmon-active substrate. Nevertheless, in this study we used 530 nm irradiation to evaluate only plasmonic activity.

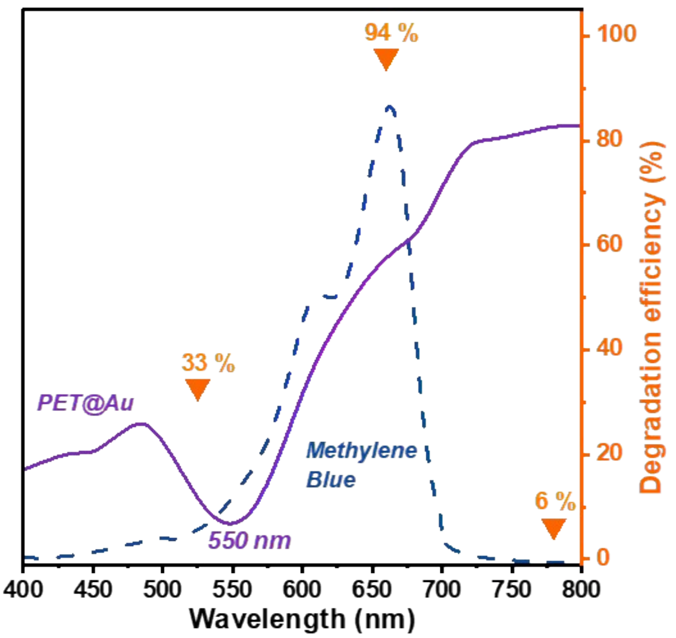

Figure S11. MB degradation efficiency under different wavelength irradiation (525, 660, 780 nm) with overlapped UV-Vis spectra of PET@Au and MB.

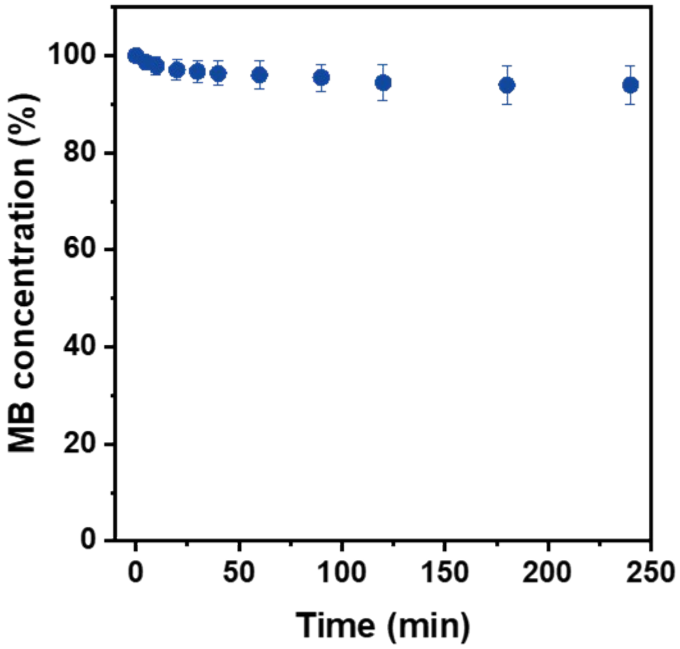

Figure S12. Kinetic curve of MB adsorption on PET@Au. The concentration of MB remains unchanged after 30 minutes of immersion, indicating that the adsorption-desorption equilibrium is reached within this time.

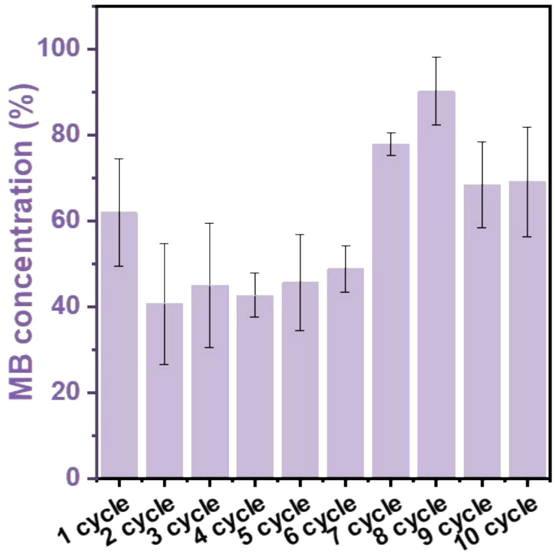

Figure S13. Results of reusability tests showing MB concentration after 10 consecutive reaction cycles using the same PET@Au.

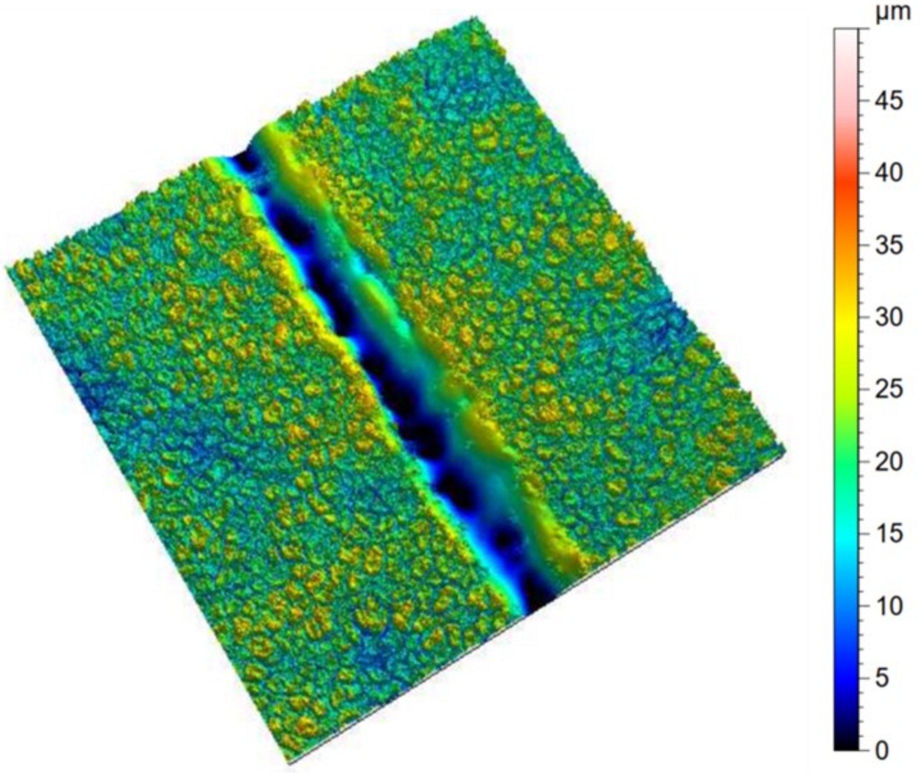

Figure S14. Confocal microscopy image of a single laser-induced Au NPs microchannel on the PET@UiO-66@Au^{3+} surface.

References:

1. J. A. Nelder and R. Mead, *The Computer Journal*, 1965, **7**, 308-313.
